# Identifying fecal microbiota signatures of colorectal cancer in a Vietnamese cohort

**DOI:** 10.3389/fmicb.2024.1388740

**Published:** 2024-12-24

**Authors:** Pham Thi Tuyet Nhung, Hang Thi Thu Le, Quang Huy Nguyen, Dao Thi Huyen, Dong Van Quyen, Le Huu Song, Tran Van Thuan, Tam Thi Thanh Tran

**Affiliations:** ^1^Hanoi Medical University, Hanoi, Vietnam; ^2^108 Military Central Hospital, Hanoi, Vietnam; ^3^Department of Life Sciences, University of Science and Technology of Hanoi, Vietnam Academy of Science and Technology, Hanoi, Vietnam; ^4^Vietnamese-German Center for Medical Research (VG-CARE), Hanoi, Vietnam; ^5^Molecular Microbiology Lab, Institute of Biotechnology (IBT), Vietnam Academy of Science and Technology (VAST), Hanoi, Vietnam

**Keywords:** colorectal cancer, fecal microbiota, cancer signatures, Vietnamese patients, 16S rRNA amplicon sequencing

## Abstract

**Background:**

Colorectal cancer (CRC) is among the top three causes of global cancer mortality. In Vietnam, CRC is the third leading cause of death in women and the fourth cause of cancer mortality in men. A large number of metagenomic studies have reported the relationship between altered composition and function of the gut microbiota with CRC, but this relationship in low- and middle-income countries including Vietnam (with an estimated population of 100.3 million people in 2023, ranking 16th largest country by population in the world) is not well-explored.

**Methods:**

We collected clinical data and fecal samples from 43 CRC patients and 44 healthy control subjects. The total community DNA of microorganisms was extracted from the fecal samples and analyzed for microbiota composition using Illumina MiSeq amplicon sequencing targeting the V3–V4 region of the 16S rRNA gene.

**Results:**

We identified a significant difference in the overall fecal microbiota composition between CRC patients and healthy controls, and we detected several CRC-associated microbial signatures in fecal samples of Vietnamese patients with CRC, which overlapped with signatures from other countries and meta-analyses. Although patients with (*n* = 8) and without (*n* = 35) type 2 diabetes (T2D) exhibited distinct gut microbiota composition compared to healthy controls, increased relative abundances of putatively pathogenic species including *Parvimonas micra, Peptostreptococcus stomatis*, and *Prevotella intermedia* were consistent biomarkers for CRC. In contrast, several health-associated species were significantly depleted in CRC patients such as *Lactobacillus johnsonii* and *Bifidobacterium longum* in CRC/non-T2D patients, *Ruminococcus s*pecies, *Bacteroides uniformis*, and *Phascolarctobacterium faecium* in CRC/T2D patients, and *Butyricicoccus pullicaecorum* in both CRC groups combined.

**Conclusion:**

Our findings confirm alterations in gut microbiota composition in CRC in a pilot Vietnamese cohort and highlight several gut microbial taxa that may have inhibitory or driver roles in CRC. This and future studies will enable the development of cancer diagnostics and treatment strategies for CRC in Vietnam, with a focus on targeting the microbiota.

## Background

Colorectal cancer (CRC) is one of the most common cancers worldwide, including in Vietnam. According to Globocan 2020, this cancer is the third most commonly diagnosed cancer and the second most lethal cancer worldwide (Sung et al., 2021). A systematic analysis of CRC from 1990 to 2017 across 195 countries revealed that the incidence and mortality rates of CRC have decreased in developed countries. This decline is attributed to the introduction of screening tests, early detection programs, and the adoption of best practices in CRC treatment and management. In contrast, the opposite trend was observed in many low-income and middle-income countries, including Vietnam (Collaborators, 2019). In Vietnam, CRC is the most common cancer of the gastrointestinal tract; the estimated number of new cancer cases in the year 2020 was more than 6,448 rectal cancer and 9,399 colon cancer, and the number of CRC deaths was reported at ~8,000 people. CRC becomes the fifth leading cause of cancer mortality in Vietnam, accounting for 9% of all new cancer cases. It is the third and fourth leading cause of cancer mortality in women and men, respectively (Sung et al., 2021). Although the majority of CRC occurs in individuals aged over 50 years, early-onset CRC (< 50 years) has increased in prevalence in recent years, suggesting as-yet-unknown environmental risk factors (Stoffel and Murphy, 2020). Apart from age-related risk, environmental factors including Western dietary habits, smoking, high intake of red meat and processed meat, heavy alcohol use, weight gain and obesity, and low physical activity can increase the risk of CRC development (Collaborators, 2019). Among the environmental risk factors, the gut microbiota plays a potential role in causing sporadic CRC. The gut microbiota is a community of microbes living in the intestine and acting as a virtual organ. Gut microbes actively interact with the colonic epithelium and contribute to maintaining gut homeostasis (Tlaskalova-Hogenova et al., 2014; Gagniere et al., 2016).

Since the majority of microbes in the human alimentary tract are present in the large intestine, many researchers are focusing on the association of the gut microbiota with intestinal carcinogenesis and its potential role in cancer treatment. Numerous studies have revealed the differences in the gut microbial composition between healthy individuals and patients with CRC and described specific bacterial markers for colorectal cancer screening (Feng et al., 2015; Baxter et al., 2016; Yachida et al., 2019). Several bacterial candidates from seven genera including *Fusobacterium, Peptostreptococcus, Porphyromonas, Prevotella, Parvimonas, Bacteroides*, and *Gemella* are associated with CRC and can potentially be used as key markers in detecting CRC (Wong and Yu, 2019; Ternes et al., 2020). Enrichment of *Gemella, Peptostreptococcus, Fusobacterium nucleatum, Leptotrichia, Selenomonas sputigena*, and *Campylobacter rectus* was observed in tumor biopsies of 43 Vietnamese CRC patients when compared to control biopsies of 25 individuals with non-cancerous colorectal polyps (Tran et al., 2022). The major challenge of gut microbiome research in humans is the normal range of differences in the composition of gut microbiota between study cohorts, between disease conditions, or even between individuals. A meta-analysis of 2,500 individuals with different diseases, including CRC, showed that the composition of the gut microbiome was most strongly influenced by regional factors such as country or continent. Additionally, the microbiome-disease signature for each condition varied from study to study (Ghosh et al., 2020). Therefore, in this pilot project, we expanded our understanding of the alteration of the gut microbiota in CRC in non-Western countries by specifically targeting the microbiota signatures in fecal samples of Vietnamese patients with CRC.

## Methods

### Study participants and clinical data collection

A total of 43 patients newly diagnosed with CRC at the 108 Military Central Hospital and 44 healthy controls from the community, who were in generally good health and showed no symptoms of CRC, were recruited for this study during the years 2022–2023. In addition, the following inclusion criteria were used for the selection of CRC and healthy subjects: aged 50–79 years, male or female, and body mass index (BMI; range: 18.5–30). If any of the following conditions applied, a participant was considered ineligible to participate in this study: complicating diseases, mental illness, evidence of a biochemical or structural abnormality of the digestive tract, irritable bowel syndrome, inflammatory bowel disease, taking any antibiotics within 4 weeks prior to recruitment, or adherence to a restricted diet.

Participants' demographic characteristics such as age, sex, BMI, health status, and medication history were collected and controlled for the data analysis. A family history of cancer and gastrointestinal and allergic disorders was also recorded. The results of blood chemistry tests and clinicopathological characteristics of the CRC patients, including tumor size, TNM (tumor, node, and metastasis) stage, tumor location, metastasis status, tumor recurrence, and survival status, were extracted from patients' medical records.

### Fecal sample collection and total genomic DNA extraction

All participants were asked to self-collect fecal samples at home or at the hospital with a plastic specimen container. Fresh feces were transported to the laboratory on dry ice to prevent bacterial growth within 4 h or were kept in cold storage (~4°C) for 24–48 h before arriving at the laboratory. Upon arrival at the laboratory, the fecal samples were subsampled into aliquots to prevent further unnecessary freeze-thaw cycles and were frozen for long-term storage at −80°C.

Total genomic DNA was extracted from the fecal samples using the Qiagen DNeasy^®^ PowerSoil^®^ Pro Kit (QIAGEN, Germany) with a bead-beating step. Briefly, 0.25 g of fecal samples were homogenized mechanically in two intervals (10 min at a speed of 25 Hz) in a PowerBead Pro Tube containing CD1 solution and beads using a TissueLyser II (QIAGEN, Germany). The subsequent steps of genomic DNA extraction were then performed according to the manufacturer's extraction instructions. Genomic DNA was measured using the NanoDrop 2000 Spectrophotometer (Thermo Fisher Scientific, USA). The genomic DNA was kept at −20°C for further use.

### Amplification and sequencing of the bacterial 16S rRNA gene

The extracted genomic DNA was used for PCR amplification with a bacterial primer pair (5′- CCTACGGRRBGCASCAGKVRVGAAT-3′ and 5′- GGACTACNVGGGTWTCTAATCC-3′) for the 16S rRNA gene V3–V4 hypervariable region. The PCR master mix contained 1 μM of each forward and reverse primer, 2 μl of dNTPs, 2.5 μl of TransStart buffer, 0.5 μL of TransStart Taq DNA polymerase, 20 ng of the extracted DNA, and 25 μl of nuclease-free water. The PCR program was as follows: 1. 94°C for 3 min, 2. 95°C for 5 s, 3. 57°C for 90 s, 4. 72°C for 10 s, and 5. 72°C for 5 min, with steps 2–4 repeated for 24 cycles. The amplicons from each sample were then incorporated with indexed adapters using limited cycle PCR and were purified using magnetic beads. The purified 16S amplicon libraries were quantified using the Tecan Infinite 200 Pro microplate reader and were pooled together at equimolar concentration. The 250 bp paired-end sequencing was processed on the pooled libraries using the Illumina MiSeq Platform (Illumina, USA) at Genewiz, Inc. (South Plainfield, NJ, USA). Raw 16S rRNA gene sequencing data is available at the NCBI Sequence Read Archive (SRA), under BioProject PRJNA1077687.

### Bioinformatics analysis of the 16S amplicon data

Analysis of 16S amplicon sequencing data was carried out as in our previous study (Tran et al., 2019). Briefly, after merging the 2x250 bp paired-end reads with FLASH v1.2.8 (Magoc and Salzberg, 2011), the merged reads were trimmed forward primers and reverse primers with cutadapt v1.8.3 (Martin, 2011) and truncate_reverse_primer.py script from Quantitative Insights Into Microbial Ecology (QIIME) v1.9.1 (Caporaso et al., 2010). The QIIME's split_libraries_fastq.py script was used to filter out low-quality reads with the quality threshold at Q19 and concatenate reads from different samples into one file. Next, the clustering of reads into operational taxonomic units (OTUs) was processed using USEARCH v8.1 software (Edgar, 2010). The reads were pre-processed using usearch -derep_fulllength, sortbylength, and sortbysize. A default threshold of 97% sequence identity was set for usearch -cluster_otus to form OTU representative sequences. Chimeras were eliminated from the OTU representative sequences using the usearch -uchime_ref with the ChimeraSlayer reference database. The usearch -usearch_global was applied to map all reads to the OTU representative sequences with an identity threshold of 97% to create the OTU table. Taxonomy assignments were obtained using mothur v1.36.1 (Schloss et al., 2009) with the Ribosomal Database Project (RDP) trainset 18 (Cole et al., 2009) and SPINGO v1.3 (Allard et al., 2015) for species classification. Alpha-diversity and beta-diversity were computed using QIIME's alpha_rarefaction.py and beta_diversity.py scripts on a rarefied OTU table at the minimum OTU reads across all samples as 70,034 reads per sample.

### Statistical analysis

Statistical analysis was performed using R v4.2.1 software (R Core Team, 2016). The significant differences in clinical measures and alpha-diversity indices were determined using the Mann–Whitney *U*-test for comparison of two independent groups and the Kruskal–Wallis test for comparison of more than two groups. Comparisons of categorical variables were calculated using Fisher's exact test for two groups and chi-squared test for >2 groups. Principal coordinate analysis (PCoA) was used to visualize the similarities in beta diversity between the samples. Permutational multivariate analysis of variance (PERMANOVA) tests were conducted to assess the significant differences in beta diversity between the groups. The differential taxonomic abundances between CRC patients and healthy individuals were calculated using DESeq2 (Love et al., 2014), controlling for age, sex, BMI, and hypertension. A *p-*value adjusted using the Benjamini–Hochberg correction, < 0.05, was considered statistically significant. The Spearman correlation between the relative abundance of OTUs and the clinical traits was conducted using the cor.test function in R.

## Results

### Clinical characteristics of study participants

The clinical descriptors of the CRC patients and healthy controls enrolled in this study are presented in Table 1. We included 43 CRC patients and 44 healthy controls, with an average age of 64 years (age range, 53–76 years) for CRC patients and an average of 61 years (age range, 51–75 years) for healthy controls. The ages were divided into three groups; the majority of healthy controls (56.8%) were 60–70 years old, while the patients with CRC were 50–60 and 60–70 years old, accounting for 65.7%. There was no significant difference between CRC patients and healthy controls with respect to BMI, sex, or family history of CRC (Mann–Whitney *U*-test, *p*-value > 0.05). A high prevalence of T2D and hypertension, which are common comorbidities in people aged over 50, was recorded in CRC patients compared to healthy individuals.

**Table 1:** Descriptive statistics of CRC patients and healthy controls.

**Characteristic**	**Categories**	**CRC patients (*n* = 43)**	**Healthy controls (*n* = 44)**	***P-*value**
Age at diagnosis	Mean ± SD (years)	64 ± 6	61 ± 6	**0.046** ^**a**^
	50–60, no. (%)	15 (34.9%)	15 (34.1%)	0.051^b^
	60–70, no. (%)	16 (30.8%)	25 (56.8%)	
	70–80, no. (%)	12 (23.1%)	4 (9.1%)	
BMI	Mean ± SD (kg/m^2^)	22.7 ± 2.2	23.2 ± 1.8	0.142^a^
	Normal weight, no. (%)	35 (67.3%)	39 (88.6%)	0.383^c^
	Overweight, no. (%)	8 (15.4%)	5 (11.4%)	
**Sex**				
	Male, no. (%)	24 (46.2%)	24 (54.5%)	0.394^c^
	Female, no. (%)	19 (36.5%)	20 (45.5%)	
**Comorbidity**				
	T2D, no. (%)	8 (15.4%)	0 (0%)	**0.002** ^**c**^
	Hypertension, no. (%)	16 (30.8%)	6 (13.6%)	**0.014** ^**c**^
Family history of CRC, no. (%)		2 (4.7%)	0 (0%)	0.241^c^

Age, weight, height, and BMI are presented with mean ± standard deviation (SD).

^a^Mann–Whitney U-test.

^b^Chi-squared test.

^c^Fisher's exact test.

Statistically significant results (p < 0.05) are shown in bold.

All patients with CRC suffered from abdominal pain. The coexistence of other symptoms included rectal bleeding, diarrhea, and/or weight loss. Among the 43 patients, five patients (11.6%) had either liver metastasis or peritoneal metastasis, while two patients (4.7%) experienced multiple organ metastases. A total of 62.5% of tumors in CRC patients were reported as 2–5 cm in size and 39.5% were 5–10 cm; all tumors were resectable. The majority of the tumors (46.5%) occurred in the left colon, followed by the right colon, the rectum, and the transverse colon, accounting for 23.3, 20.9, and 9.3%, respectively. According to the TNM classification, the recruited patients ranged from early to advanced stages, with the majority (72.1%) being in stages II and III. Cancer recurred in 11.6% of the patients, and three patients (7%) died within 2 years of diagnosis (Table 2). In addition to clinical features, blood biochemical characteristics were investigated in CRC patients. The mean values of most parameters in both male and female CRC patients were within the normal range. This indicates that CRC itself was not the cause of the blood test changes. On the contrary, carcinoembryonic antigen (CEA) level, a prognostic marker for CRC, was elevated in the serum of 21 patients (48.8%), especially patients with metastases (Supplementary Table 1).

**Table 2:** Clinicopathological characteristics of CRC patients.

**Characteristic**	**Categories**	**CRC patients (*n* = 43)**
Tumor size (cm)	Median (Range)	4.5 (2–10)
	2–5 cm	26 (60.5%)
	5–10 cm	17 (39.5%)
**TNM stage** ^*^ **, no. (%)**		
	Stage I	7 (16.3%)
	Stage II	13 (30.2%)
	Stage III	18 (41.9%)
	Stage IV	5 (11.6%)
**Tumor location, no. (%)**		
	Left colon	20 (46.5%)
	Right colon	10 (23.3%)
	Transverse colon	4 (9.3%)
	Rectum	9 (20.9%)
**Metastasis, no. (%)**		
	One organ	5 (11.6%)
	>1 organ	2 (4.7%)
Tumor recurrence, no. (%)		5 (11.6%)
**Survival status, no. (%)**		
	Living	40 (93%)
	Dead	3 (7%)

^*^TNM stage: Tumor, node, and metastasis (TNM) stage.

### Gut microbiota diversity in CRC patients and healthy controls

**Figure 1:**
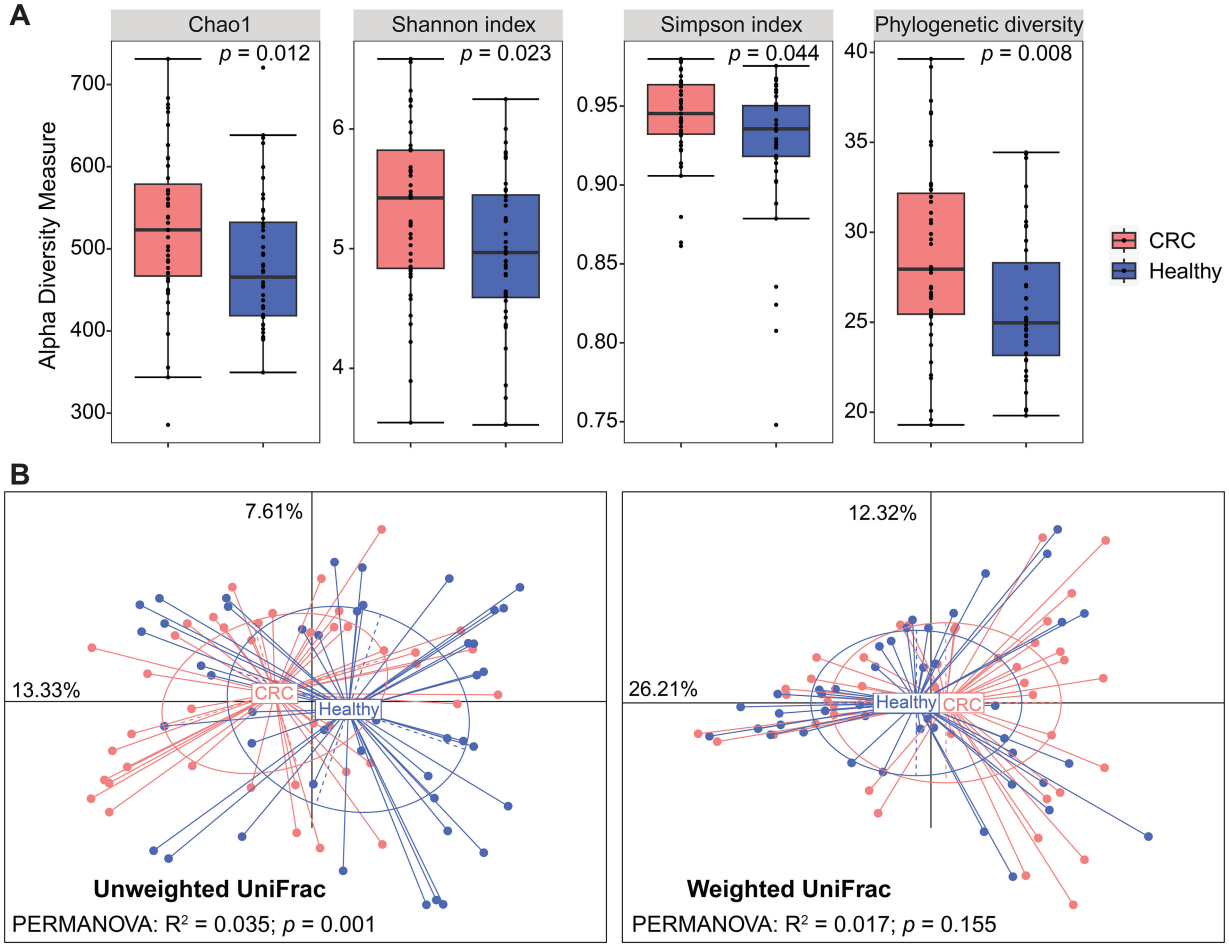
Diversity of the gut bacterial communities differs between CRC patients and healthy controls. **(A)** Boxplots of four alpha-diversity indices visualize the difference in the species richness and evenness of CRC patients compared to healthy controls. *P*-values were determined using the Mann–Whitney *U*-test. **(B)** Principal coordinate analysis (PCoA) plots based on the unweighted and weighted UniFrac distances. Permutational multivariate analysis of variance (PERMANOVA) tests were conducted to assess significant differences between CRC and healthy groups.

Total genomic DNA was extracted from fecal samples of individual participants and underwent 16S rRNA gene amplicon sequencing for the identification of bacterial diversity and composition. We observed a significant increase in alpha diversity measured using the Chao1, Shannon's, Simpson's, and phylogenetic diversity indices in Vietnamese patients with CRC compared to healthy controls (Mann–Whitney *U*-test, *p*-value < 0.05; Figure 1A). The alpha-diversity indices did not differ by the participant's age category (50–60, 60–70 vs. 70–80 years), BMI category (normal weight vs. overweight), sex (male vs. female), and blood pressure (hypertension vs. non-hypertension), although the Simpson's index was slightly lower in the participants with T2D (median = 0.93; IQR = 0.90–0.94) than the participants without T2D (median = 0.94; IQR = 0.93–0.96; *p*-value = 0.098). Furthermore, alpha diversity in the CRC group was not influenced by any clinical traits, except for the BMI category and T2D status. In contrast, the Chao1 index differed among age categories in the healthy control group (Supplementary Table 2).

Upon assessment of the overall gut microbiota composition, beta diversity represented by unweighted UniFrac distance revealed two fairly distinct clusters based on CRC condition (PERMANOVA: R^2^ = 0.035; *p* = 0.001), whereas no significance was detected between CRC patients and healthy controls based on the weighted UniFrac distance (PERMANOVA: R^2^ = 0.017; *p* = 0.155; Figure 1B). In addition, there were no significant differences by the participant's age category, BMI category, sex, T2D condition, or blood pressure in either UniFrac distance. However, unweighted and weighted UniFrac distances were discriminatory in terms of BMI categories in the CRC cohort, with a low coefficient of determination (PERMANOVA: R^2^ = 0.037 and 0.058, respectively; *p* < 0.05 for both; Supplementary Figure 1A and Table 3). Within the healthy subjects' samples, there was a significant separation among age categories based on the weighted UniFrac distance (PERMANOVA: R^2^ = 0.098; *p* = 0.01), and sex had a relevant impact on the weighted UniFrac distance (PERMANOVA: R^2^ = 0.043; *p* = 0.015; Supplementary Figure 1B and Table 3).

**Table 3:** Association between beta-diversity and clinical characteristics.

	**Unweighted UniFrac**		**Weighted UniFrac**	
	***R** ^2^*	***p***	***R** ^2^*	***p***
**All samples (CRC and healthy samples)**				
*CRC condition*: CRC vs. healthy	0.035	**0.001**	0.017	0.155
*Age category*: 50–60, 60–70 vs. 70–80	0.025	0.267	0.026	0.292
*BMI category*: normal weight vs. overweight	0.014	0.152	0.009	0.617
*Sex*: male vs. female	0.015	0.103	0.011	0.462
*T2D condition*: T2D vs. non-T2D	0.012	0.371	0.012	0.362
*Blood pressure*: hypertension vs. non-hypertension	0.011	0.539	0.006	0.897
**CRC samples**				
*Age category*: 50–60, 60–70 vs. 70–80	0.045	0.580	0.066	0.121
*BMI category*: normal weight vs. overweight	0.037	**0.032**	0.058	**0.017**
*Sex*: male vs. female	0.020	0.744	0.014	0.821
*T2D condition*: T2D vs. non-T2D	0.027	0.248	0.032	0.198
*Blood pressure*: hypertension vs. non-hypertension	0.020	0.722	0.006	0.994
*Tumor size*: 2–5 cm vs. 5–10 cm	0.024	0.428	0.019	0.626
*TNM stage*: four stages	0.066	0.700	0.074	0.413
*Tumor location*: four locations	0.069	0.562	0.062	0.640
*Metastasis*: yes vs. no	0.021	0.661	0.023	0.426
*Tumor recurrence*: yes vs. no	0.019	0.810	0.012	0.906
*Survival status*: living vs. dead	0.021	0.630	0.014	0.821
*CEA*: CEA-normal vs. CEA-elevated	0.022	0.624	0.025	0.381
**Healthy samples**				
*Age category*: 50–60, 60–70 vs. 70–80	0.054	0.176	0.098	**0.010**
*BMI category*: normal weight vs. overweight	0.021	0.645	0.035	0.144
*Sex*: male vs. female	0.043	**0.015**	0.019	0.627
*Blood pressure*: hypertension vs. non-hypertension	0.025	0.327	0.028	0.261

R^2^ and p-values were obtained from permutational multivariate analysis of variance (PERMANOVA) tests. Statistically significant (p < 0.05) is shown in bold.

### Intestinal microbial associations with colorectal cancer

**Figure 2:**
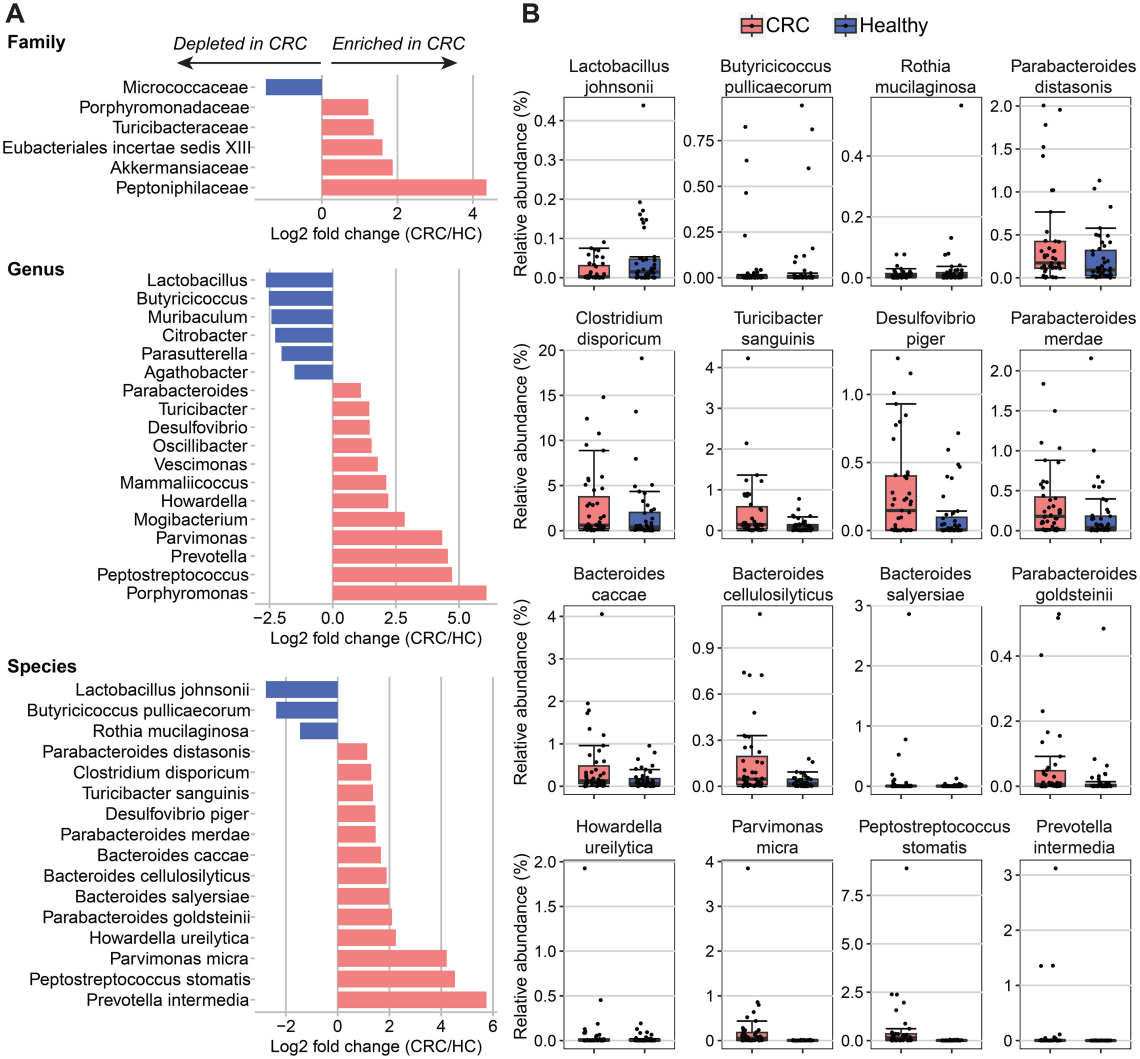
Differences of bacterial taxa abundance between CRC patients and healthy controls (HC). **(A)** Fold changes in different taxa levels (family, genus, and species) correspond to differences in all CRC patients (*n* = 43) compared to healthy controls (*n* = 44). Differential abundances were calculated using DESeq2, controlling for age, sex, BMI, and hypertension. A significant positive fold change (pink color bar) represents an increase of taxa in patients with CRC compared with healthy control, whereas a blue color bar shows the opposite direction. **(B)** Boxplots of significantly different species between all CRC patients and healthy controls.

We observed high variability in microbiome composition at the family and genus levels among individuals in both the CRC and control groups. The most dominant families in the participants' gut were *Lachnospiraceae, Oscillospiraceae, Bacteroidaceae, Enterobacteriaceae*, and *Peptostreptococcaceae*, with a mean relative abundance of 74.6% (range = 44.01–82.18%, SD = 11.24; Supplementary Figure 2A). At the genus level, the relative abundance means of the top five genera were 16.81% (SD = 10.63) for *Blautia*, 5.69% (SD = 4.83) for *Faecalibacterium*, 4.68% (SD = 6.22) for *Phocaeicola*, 4.21% (SD = 7.46) for *Escherichia/Shigella*, and 4% (SD = 5.05) for *Bacteroides* (Supplementary Figure 2B). Next, we identified 6 classified families and 18 classified genera that significantly correlated with the CRC condition using DESeq2, controlling for age, sex, BMI, and hypertension (Figure 2A). Compared to the healthy individuals, *Porphyromonadaceae, Turicibacteraceae, Eubacteriales incertae sedis* XIII, *Akkermansiaceae*, and *Peptoniphilaceae* were found to be increased in CRC samples, while *Micrococcaceae* was negatively associated with CRC condition. Similarly, the genera *Agathobacter, Parasutterella, Citrobacter, Muribaculum, Butyricicoccus*, and *Lactobacillus* were underrepresented in the CRC patients. On the contrary, 12 out of 18 genera were positively associated with CRC, with the top genera showing the highest log2 fold changes in abundance in CRC compared to healthy controls, namely *Mogibacterium, Porphyromonas, Peptostreptococcus, Prevotella*, and *Parvimonas* (log2 fold change > 2.5, adjusted *p-*value < 0.001; Figure 2A and Supplementary Table 3).

A comparison of bacterial species detected a lower abundance of three species and a higher abundance of 13 species in the CRC patients than in the healthy controls (Figure 2 and Supplementary Table 4). Among those, *Parvimonas micra, Peptostreptococcus stomatis*, and *Prevotella intermedia* showed the greatest variance between the CRC and healthy groups (log2 fold change > 4, adjusted *p-*value < 0.001). In addition, we found significant enrichment of three *Bacteroides (B.)* species (*B. caccae, B. cellulosilyticus*, and *B. salyersiae)* and *three Parabacteroides (P.)* species (*P. distasonis, P. merdae*, and *P. goldsteinii*) in patients with CRC. On the contrary, CRC patients had lower abundance levels of two health-associated species including *Lactobacillus johnsonii* and *Butyricicoccus pullicaecorum* than the healthy individuals (log2 fold change < −2, adjusted *p-*value < 0.05).

### Impact of type 2 diabetes on fecal microbiota signatures of colorectal cancer

**Figure 3:**
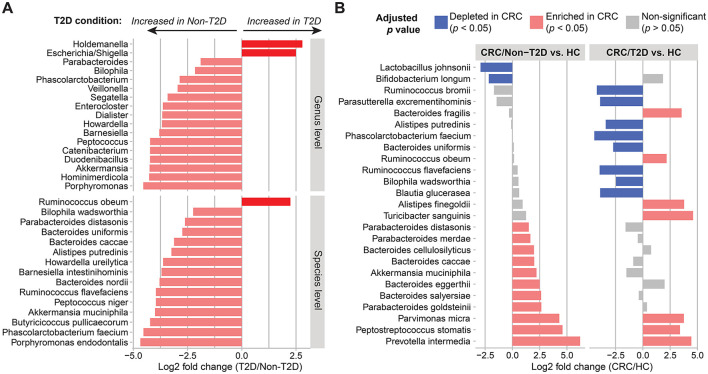
Alterations of gut microbiota composition in the presence of CRC and T2D. **(A)** Genus- and species-level gut composition in CRC patients differed based on the co-existence of T2D. **(B)** Differences in species abundance in CRC patients without (*n* = 35) and with T2D (*n* = 8) compared to healthy controls (HC; *n* = 44). Differential abundances between the two groups were calculated using DESeq2, controlling for age, sex, BMI, and hypertension. A significant positive fold change (pink color bar) represents an increase of taxa in patients with CRC compared with healthy control, whereas a blue color bar shows the opposite direction.

T2D is a common comorbidity in patients with CRC, and this metabolic disease has been reported to be associated with alteration in the gut microbiota community (Umirah et al., 2021). Thus, we further evaluated CRC-related gut microbiota signatures in the presence of T2D. A comparison between CRC patients without T2D (*n* = 35) and those with T2D (*n* = 8) revealed significant differences in 17 genera and 15 species (Figure 3A). Among the significantly associated species, the top six species that showed the most significant fold change between the two groups were *Ruminococcus flavefaciens, Peptococcus niger, Akkermansia muciniphila, Butyricicoccus pullicaecorum, Phascolarctobacterium faecium*, and *Porphyromonas endodontalis* (log2 fold change > 3.9, adjusted *p-*value < 0.05; Figure 3A and Supplementary Figure 3).

Next, in comparison to the healthy control group, the families *Turicibacteraceae* and *Peptoniphilaceae* and six genera including *Turicibacter, Mogibacterium, Parvimonas, Prevotella, Peptostreptococcus*, and *Porphyromonas* were consistently enriched in CRC patients with and without T2D (log2 fold change > 1, adjusted *p-*value < 0.05; Supplementary Figure 4). At the species level, only *Parvimonas micra, Peptostreptococcus stomatis*, and *Prevotella intermedia* were associated with CRC patients with either non-T2D or T2D. On the other hand, the potential health-associated species were not shared between CRC/non-T2D and CRC/T2D. For example, the two species *Lactobacillus johnsonii* and *Bifidobacterium longum* were significantly increased in healthy individuals when compared to CRC/non-T2D. Meanwhile, the combination of CRC and T2D caused the change in the addition of species, increasing *Turicibacter sanguinis, Alistipes finegoldii, Bacteroides fragilis*, and *Ruminococcus obeum* and reducing *Phascolarctobacterium faecium, Ruminococcus bromii, Ruminococcus flavefaciens, Parasutterella excrementihominis, Blautia glucerasea, Alistipes putredinis, Bacteroides uniformis, and Bilophila wadsworthia* (Figure 3B and Supplementary Table 4). However, a larger sample size for the CRC/T2D group would be needed to confirm this finding. Together, these results suggested that both CRC and T2D contributed significantly to the alterations of the gut microbiota composition.

### Correlation of gut microbial taxa and clinical traits in CRC patients

**Figure 4:**
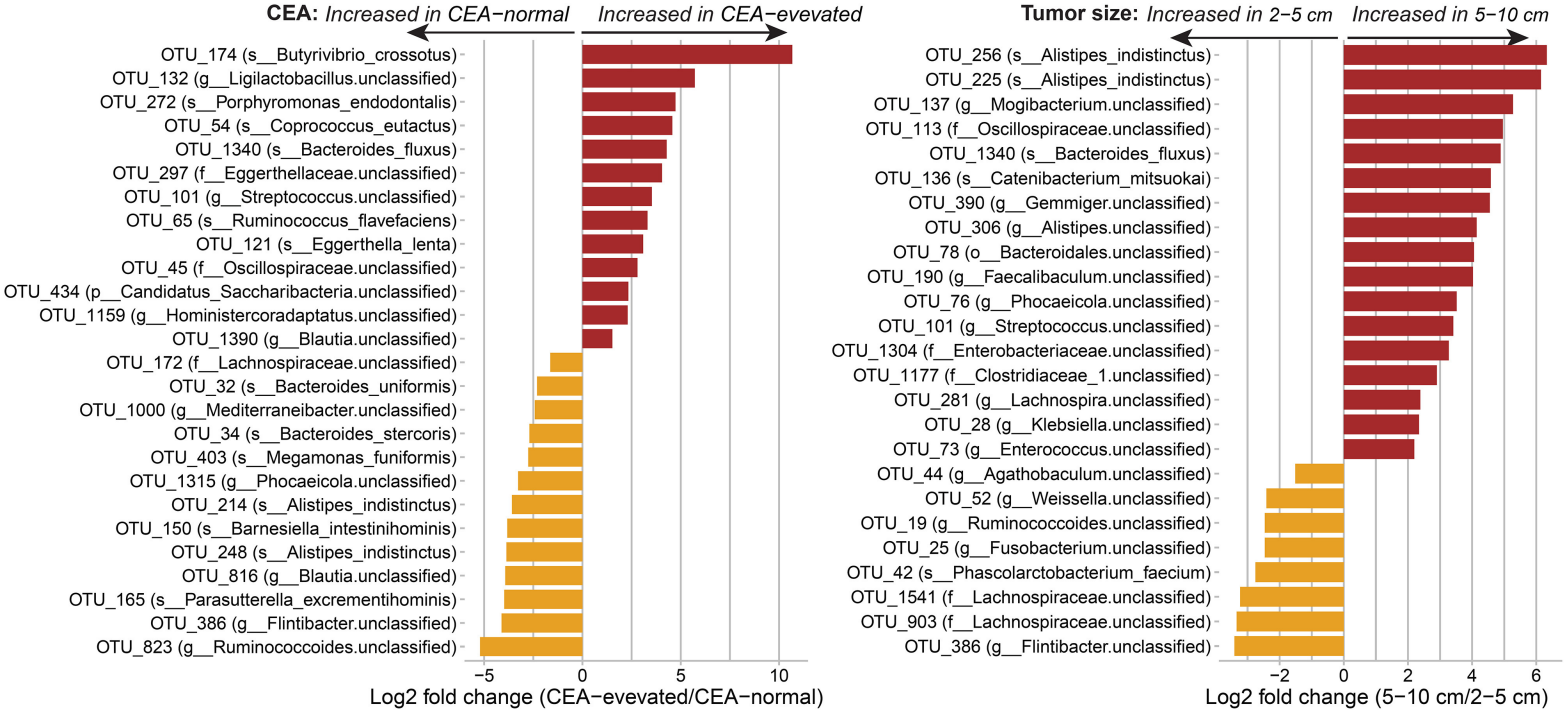
Associations of the human gut of CRC patients with the carcinoembryonic antigen (CEA) and the tumor size at the OTU level. Differential abundances were calculated using DESeq2, controlling for age, sex, BMI, hypertension, and T2D condition. Only significant taxa with adjusted *p*-value < 0.05 are shown. OTUs are labeled with species name (s___), or with genus (g___), family (f___), or phylum (p___) for unclassified species.

We examined whether the composition of the human gut of CRC patients differed based on clinical traits such as CEA level and tumor size. Since the T2D condition was found to be correlated with changes in the gut composition in CRC patients, we adjusted for the presence of T2D along with age, sex, BMI, and hypertension when conducting the differential abundance analysis of the gut microbiota for the level of CEA and the tumor size using DESeq2. Overall, 26 OTUs showed significant variation between the CEA-normal and CEA-elevated groups, while 25 OTUs were significantly associated with the tumor size (Figure 4). The CEA level and the tumor size shared three common microbial OTUs: OTU_1340, belonging to the species *Bacteroides fluxus*; OTU_101 and OTU_386, belonging to the genus *Streptococcus* and the genus *Flintibacter*, respectively.

Next, the Spearman correlation analysis in the CRC subgroups (CRC/non-T2D and CRC/T2D) revealed the differences in OTUs correlated with the CEA level and the tumor size (Supplementary Figure 5 and Supplementary Table 5). We observed strong correlations between microbial OTUs and the CEA level and the tumor size in the CRC/T2D group (Spearman's correlation coefficient, 0.7 < |ρ| < 0.9; nominal *p*-values < 0.05); meanwhile, moderate relationships were shown in the CRC/non-T2D group (Spearman's correlation coefficient, 0.3 < |ρ| < 0.6; nominal *p*-values < 0.05). Specifically, the relative abundances of several OTUs belonging to the *Lachnospiraceae* were inversely correlated with tumor size in both CRC/non-T2D (OTU_1275, OTU_1278, and OTU_1541) and CRC/T2D (OTU_83, OTU_176, OTU_385, OTU_1104, OTU_1198, and OTU_1541). Furthermore, the OTUs that correlated with either the CEA level or the tumor size were separated into three distinct clusters by the hierarchical cluster analysis. Cluster 1 was composed of five out of a total of 9 OTUs that were positively associated with the tumor size in CRC/T2D samples, whereas the majority of negative associations with CEA level and tumor size were observed in cluster 2 and cluster 3 in both the CRC subgroups, except for the association between the CEA level and its correlated OTUs in the CRC/T2D group. However, the microbial abundance heatmap of the correlated OTUs showed no clear separation of CRC samples according to TNM stage, tumor location, and metastasis (Supplementary Figure 5).

## Discussion

We present the fecal microbiota profiles from Vietnamese patients diagnosed with CRC compared to healthy individuals using 16S rRNA amplicon sequencing. We also extended the investigation between the gut microbiota profiles and various clinical characteristics including age, BMI, sex, comorbidities (T2D and hypertension), and clinicopathological characteristics in the CRC group. Compared to other clinical characteristics, the CRC condition is the main factor that drives the divergence of the gut microbiota diversity and composition in this study cohort.

Increased alpha diversity, species richness, and evenness were observed in CRC patients compared to healthy controls. Several previous studies reported a significantly lower alpha diversity related to CRC patients (Ahn et al., 2013; Ai et al., 2019), but contradictory results were also observed (Young et al., 2021). We hypothesized that the presence of multiple pathogenic bacteria in our CRC patients may contribute to higher alpha diversity. Further research in a large sample size is needed to identify precisely whether alpha diversity is negatively or positively correlated with CRC. In the present study, CRC condition along with BMI, age, and sex was found to be associated with the variability of the overall gut microbiota composition, in line with previous reports showing divergence of gut microbial profiles depending on host clinical features (Ghosh et al., 2020). Therefore, adjustments for potential confounders such as age, sex, BMI, and hypertension were implemented when comparing different microbial taxa between CRC patients and healthy controls.

A comparison of our findings with several published studies across various cohorts revealed both global and regional CRC-associated taxonomic signatures at the species level (Supplementary Table 6). In particular, we observed the over-representation of a set of global microbial signatures including *Parvimonas micra, Peptostreptococcus stomatis*, and *Prevotella intermedia* species in the CRC patients with and without T2D in comparison with the healthy individuals in our study. The presence of *Parvimonas micra* is associated with the upregulation of miR-218-5p, which inhibits the expression of protein tyrosine phosphatase receptor, thereby activating the Ras/ERK/c-Fos pathway and promoting CRC cell proliferation (Chang et al., 2023). Members of the genus *Peptostreptococcus, Peptostreptococcus stomatis* are two of the four universal fecal microbial signatures for CRC that have been found in Chinese, Danish, Austrian and French cohorts (Yu et al., 2017). *Prevotella intermedia* has been described to be more abundant in colorectal adenocarcinoma tissues as well as in the fecal samples of CRC patients, which may promote migration and invasion of CRC tumor cells (Avuthu and Guda, 2022; Lo et al., 2022; Tito et al., 2024). In addition, the relative dominance of *Bacteroides* and *Parabacteroides* species (*B. caccae, B. cellulosilyticus, B. salyersiae, P. distasonis, P. merdae*, and *P. goldsteinii)* was found in the CRC patients without T2D, which partly resembled previous findings (Feng et al., 2015; Obon-Santacana et al., 2022; Iadsee et al., 2023). These *Parabacteroides* species could be regarded as regional-specific microbial signatures, as they were present in only a few Asian cohorts. For example, *Parabacteroides distasonis* was observed in the Vietnamese cohort (our study) as well as in Thai and Indian cohorts (Gupta et al., 2019; Iadsee et al., 2023). Meanwhile, discrepancies in *Bacteroides* species except *Bacteroides fragilis* were noted across different study cohorts (Supplementary Table 6), suggesting that these signatures may represent country-specific microbial signatures. Both beneficial and pathogenic roles are reported for *Bacteroides* and *Parabacteroides* species (Ezeji et al., 2021; Zafar and Saier, 2021). For example, colonic mucus-degrading bacteria such as *B. caccae* were positively associated with a low-fiber diet, and they apparently obtain an energy source by degrading the intestinal mucus barrier, which could lead to increased pathogen susceptibility (Desai et al., 2016). Similarly, even though *Parabacteroides* species such as *Parabacteroides goldsteinii* are considered novel next-generation probiotics (Wu et al., 2019), they can act as opportunistic pathogens that are positively correlated with the early stage of Crohn's disease and Lynch syndrome (Mori et al., 2019; Ma et al., 2022). *Parabacteroides distasonis* has been shown to exert a protective role by suppressing colon tumorigenesis and maintaining the intestinal epithelial barrier in a murine model (Koh et al., 2020). Since this species was one of the CRC-enriched species in the CRC/non-T2D patients, it was not considered for anti-cancer properties in this study. Nevertheless, we observed an over-representation of this species in the CRC patients without T2D compared to the CRC patients with T2D, suggesting a protective effect against T2D in CRC patients. Among the enriched species that were reported in the CRC/T2D patients compared to the healthy group, we also found a *Bacteroides* species, *Bacteroides fragilis*. The association between *Bacteroides fragilis* and the induction of interleukin-17, which leads to colonic tumor formation, has been reported in a mouse model (Wu et al., 2009). In our study cohort, we did not detect the prevalence of the opportunistic pathogen *Fusobacterium nucleatum* in either CRC patients with or without T2D. This species is widely regarded as a biomarker for CRC in many cohorts as well as in another Vietnamese cohort study (Tran et al., 2022). We acknowledge that the study by Tran et al. (2022) and the current study differed in several aspects, including the CRC patient cohorts from two different regions of Vietnam, sample collection times, sample types, genomic DNA extraction, and sequencing platforms. Thus, additional validation studies are required to confirm specific fecal microbial biomarkers for the early screening of CRC.

In contrast, a decreasing trend was found for some health-associated species including *Lactobacillus johnsonii* and *Bifidobacterium longum* in the CRC/non-T2D samples and *Butyricicoccus pullicaecorum* in both CRC patients with/without T2D combined, which highlight the potential importance of health-associated species for human gut health. Indeed, strains of *Lactobacillus johnsonii*, recognized as a potential probiotic of the genus *Lactobacillus*, have been shown to have multiple beneficial effects such as modulating innate immune response and enhancing gut barrier integrity (Zheng et al., 2021; Zhang et al., 2023). The anti-CRC potential of the butyrate-producing *Butyricicoccus pullicaecorum* and the acetate-producing *Bifidobacterium longum* has been clinically demonstrated (Chang et al., 2020; Liang et al., 2023). Short-chain fatty acids (SCFAs) such as acetate and butyrate produced by those bacteria are reported to reinforce the intestinal defense of host epithelial cells and to regulate the immune response by a process involving regulatory T cells and IL-10-producing T cells (Fukuda et al., 2011; Singh et al., 2014). Unlike the gut microbiota profiles of CRC patients without T2D, *Phascolarctobacterium faecium, Ruminococcus bromii, Ruminococcus flavefaciens, Parasutterella excrementihominis, Blautia glucerasea, Alistipes putredinis, Bacteroides uniformis*, and *Bilophila wadsworthia* were depleted in CRC/T2D patients. Previous research presented a significant decrease in the genera *Bacteroides* and *Parasutterella* in CRC patients compared to healthy controls (Wang et al., 2012). In addition, Li L. et al. reported that *Bacteroides uniformis* and *Phascolarctobacterium faecium* were negatively associated with the T2D phenotype and were considered to play a pivotal role in the regulation of insulin secretion and glucose metabolism (Li et al., 2020). The genus *Ruminococcus*, including *Ruminococcus bromii* and *Ruminococcus flavefaciens*, is widely known for the degradation of resistant starch, producing SCFAs (Chassard et al., 2010; Ze et al., 2012).

While the current study provides insight into the association between gut microbiota profiles and CRC patients in Vietnamese cohorts, there are some limitations. First, the study cohort enrolled participants in north and north-central coastal regions, which does not represent gut microbiota profiles for all the different geographical regions of Vietnam. It would be interesting to investigate if any fecal microbiota biomarkers for CRC are common or unique to specific regions in Vietnam. Second, on average, the age of the healthy control group was lower than that of the CRC group because the study participants, especially the healthy individuals, were randomly selected within a wide age range (aged 50–79 years) and younger people were more likely associated with better health. To rectify this, differences in the gut microbial taxa were adjusted for the age factor for all analyses by linear models implemented in DeSeq2. Third, although a modest sample size was recruited for this pilot study, statistical power remained inadequate when studying correlations between the gut microbial taxa and clinical traits in CRC patients. Finally, we have not investigated the association between CRC and the functional capacity of the gut microbiota due to the limitations of 16S rRNA gene amplicon sequencing. A larger study in both gut microbiota composition and functional profiles, ideally sampling from multiple regions that capture gut microbiota diversity across different geographical regions of Vietnam, will ultimately provide a comprehensive picture for fully understanding the dynamic interaction of the gut microbiota and clinical characteristics of CRC patients.

## Conclusion

The current study provided new insights into the alterations of the gut microbiota composition and fecal microbial signatures associated with Vietnamese patients diagnosed with CRC. These findings lay the foundations for the application of next-generation sequencing technology in early CRC detection and the use of personalized microbiota-targeted therapy for the prevention and treatment of CRC in Vietnam.

## Data Availability

The datasets presented in this study can be found in online repositories. The names of the repository/repositories and accession number(s) can be found in the article/Supplementary material.
