# Supplementary material for: Identifying fecal microbiota signatures of colorectal cancer in a Vietnamese cohort
Source: Front Microbiol. 2024 Dec 24;15:1388740. doi: 10.3389/fmicb.2024.1388740 (PMC11704495; doi:10.3389/fmicb.2024.1388740)
Supplement: Supplementary file 2 [file Data_Sheet_1.pdf]

A

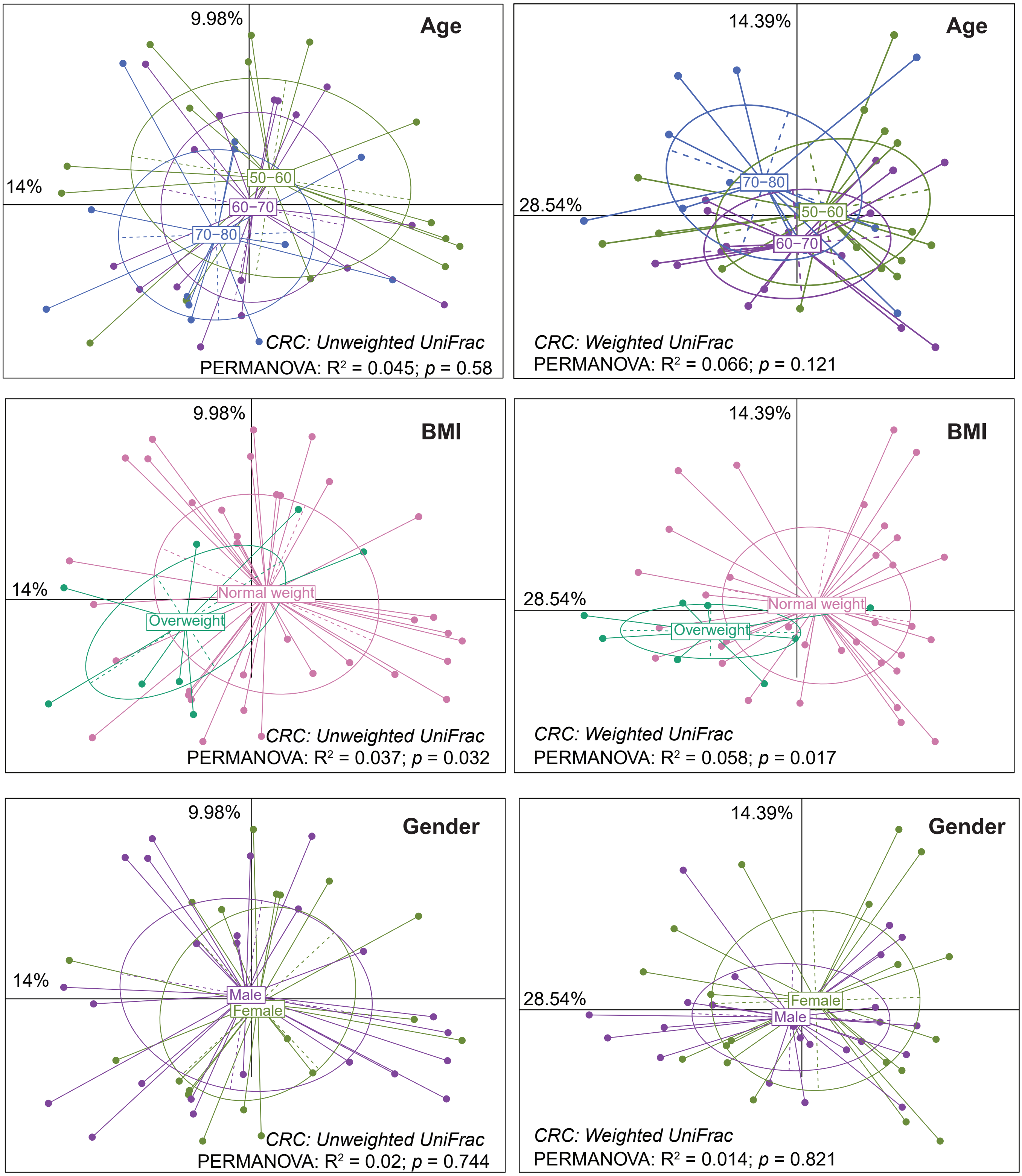
9.98%
Age
14%
50−60
60−70
70−80
CRC: Unweighted UniFrac
PERMANOVA: R² = 0.045; p = 0.58
14.39%
Age
28.54%
70−80
50−60
60−70
CRC: Weighted UniFrac
PERMANOVA: R² = 0.066; p = 0.121
9.98%
BMI
14%
Normal weight
Overweight
CRC: Unweighted UniFrac
PERMANOVA: R² = 0.037; p = 0.032
14.39%
BMI
28.54%
Normal weight
Overweight
CRC: Weighted UniFrac
PERMANOVA: R² = 0.058; p = 0.017
9.98%
Gender
14%
Male
Female
CRC: Unweighted UniFrac
PERMANOVA: R² = 0.02; p = 0.744
14.39%
Gender
28.54%
Female
Male
CRC: Weighted UniFrac
PERMANOVA: R² = 0.014; p = 0.821

B

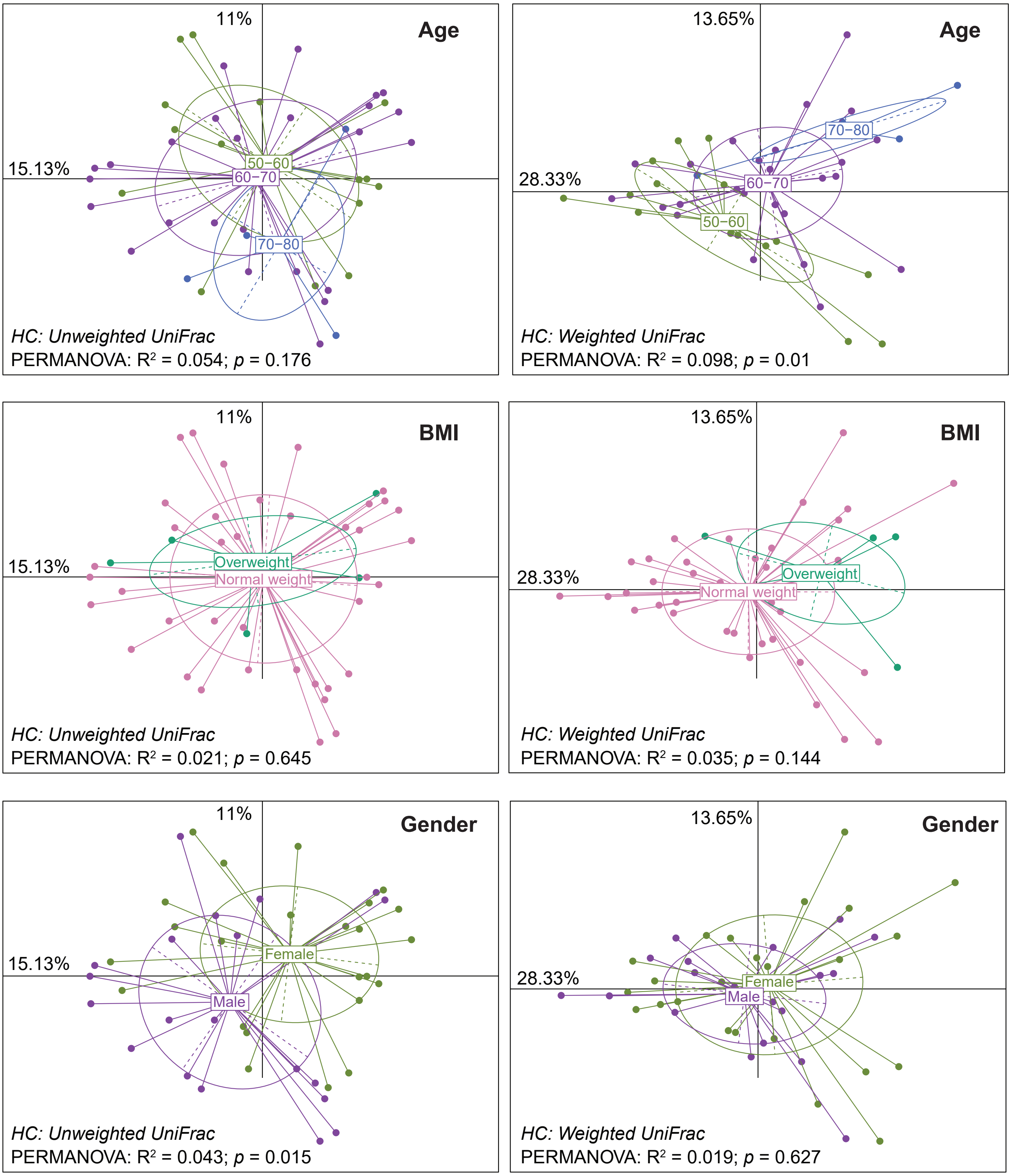

Figure S1. Principle coordinates analysis (PCoA) plots based on the Unweighted and Weighted UniFrac distances grouped by Age, BMI and Gender in CRC samples (**A**) and Healthy controls (**B**). Permutational multivariate analysis of variance (PERMANOVA) tests were conducted to access significant differences between groups.

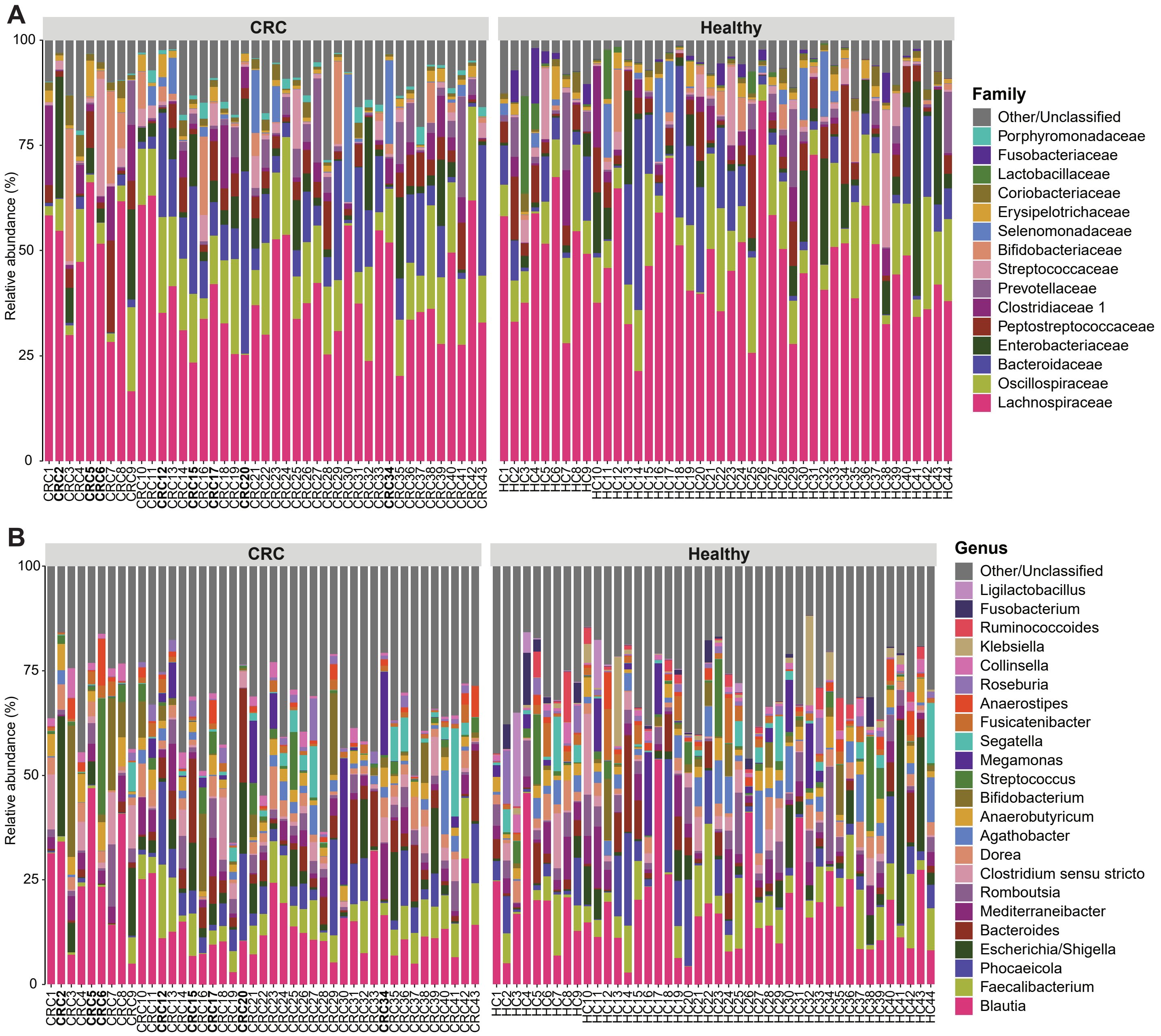

Figure S2. Taxonomic abundance of the gut bacterial communities in CRC and Healthy samples at family (**A**) and genus (**B**) levels. Only the known taxa with mean relative abundance above 1% were presented in bar plots and "Other/Unclassified" indicated the rest of taxa. CRC patients with T2D were highlighted by bold text.

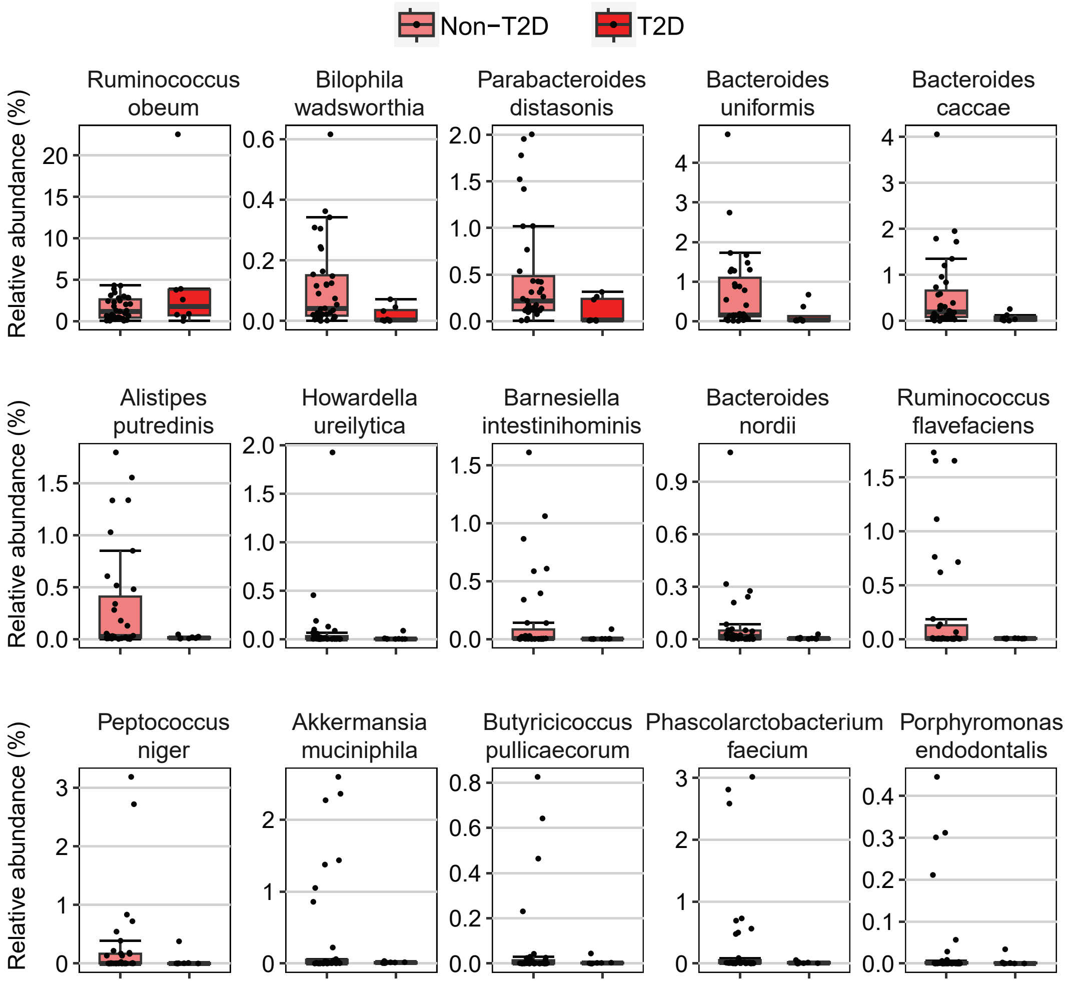

Figure S3. Boxplots of significantly different species between CRC patients without T2D and CRC patients. Differential taxonomic abundances between CRC/Non-T2D and CRC/T2D groups were calculated with DESeq2. Only the significant species (adjusted p value < 0.05) were shown.

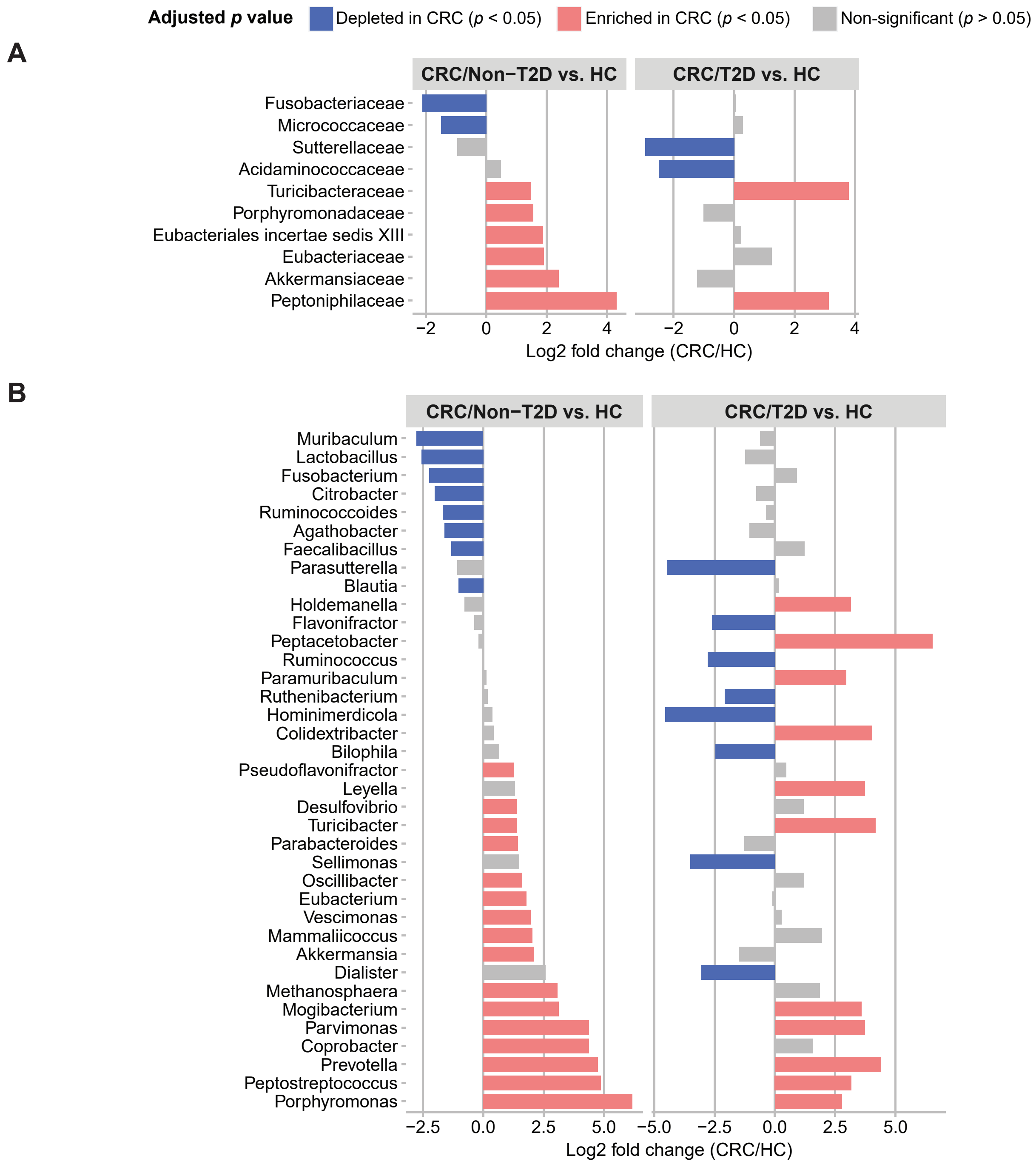

Figure S4. Differences of family (**A**) and genus (**B**) abundance in CRC patients without (n = 35) and with T2D (n = 8) compared to Healthy controls (HC) (n = 44). Differential abundances between two groups were calculated with DESeq2 controlling for age, gender, BMI and hypertension. A significant positive fold change (pink color bar) represents an increase of taxa in patients with CRC compared with healthy control, whereas a blue color bar shows opposite direction.

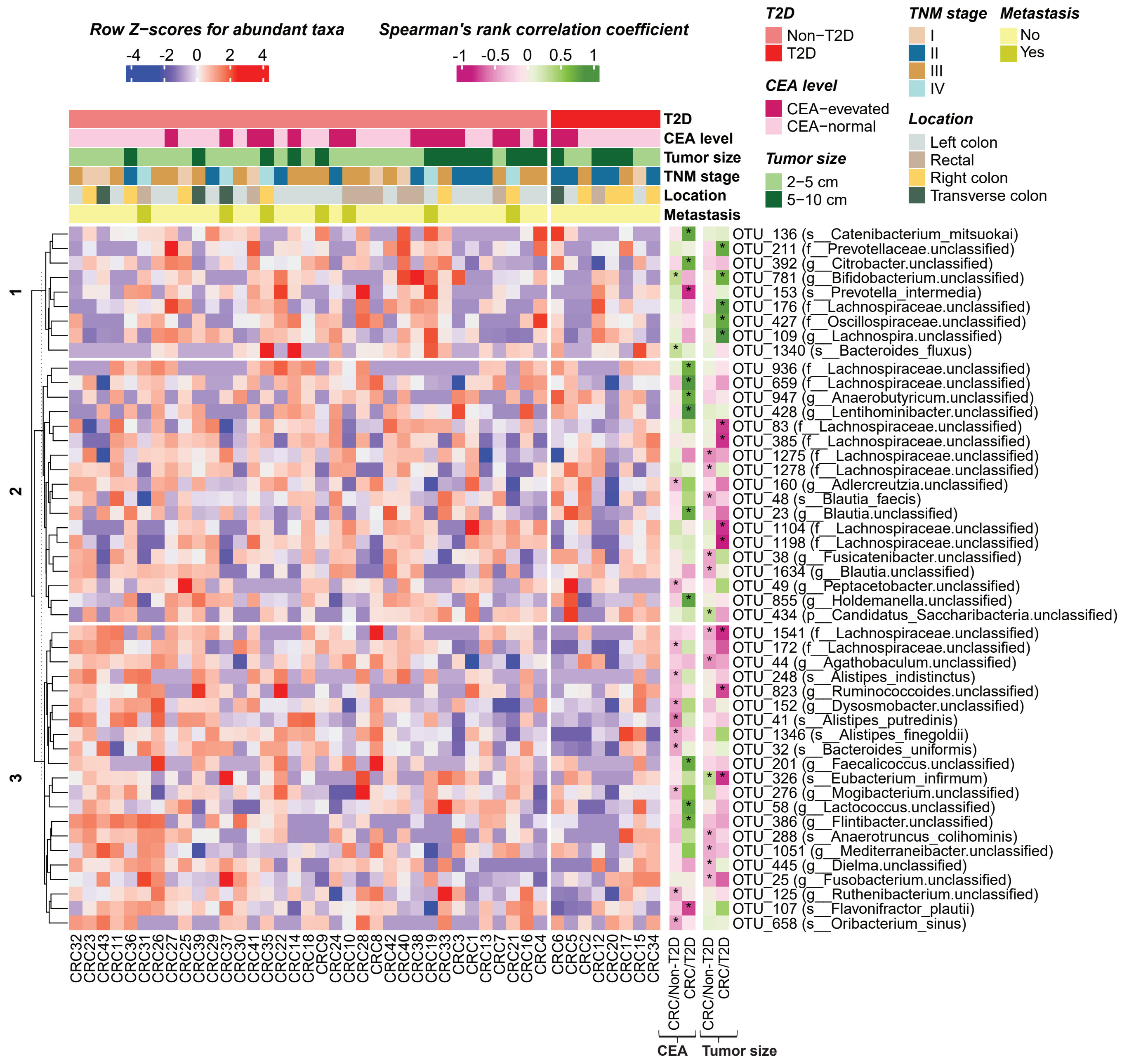

Figure S5. Heatmap of the relative abundances of operational taxonomic units (OTUs) in CRC patients without T2D and CRC patients with T2D that were associated with the carcinoembryonic antigen (CEA) or the tumor size. The hierarchical cluster analysis of OTUs in the right heatmap was obtained with Euclidean distance and Ward hierarchical algorithm. The color intensity from blue to red was linear correlated with Z-scores which indicated normalization values of the relative abundances of OTU. The Spearman correlation between the relative abundance of OTUs and the clinical traits (the CEA level and tumor size) in CRC/non-T2D and CRC/T2D subgroups were present in the left heatmap with the color intensity ranged from purple to green. Significant correlation with nominal p value < 0.05 indicated by an asterisk (*). The color bars at the top of the heatmap presented clinical characteristics of patients including T2D condition, the level of CEA, tumor size, TNM stage, location and metastasis.
